# Non-steroidal anti-inflammatory drugs improve bovine blastocyst formation and quality in in vitro culture

**DOI:** 10.5194/aab-68-555-2025

**Published:** 2025-09-11

**Authors:** Kübra Karakaş Alkan, Fatma Satılmış, Yunus Emre Deniz, Mustafa Bodu, Muhammed Furkan Çiftçi, Ömer Faruk Yeşilkaya, Hasan Alkan

**Affiliations:** 1 Department of Obstetrics and Gynecology, Faculty of Veterinary Medicine, Selcuk University, Konya, Türkiye; 2 Department of Reproduction and Artificial Insemination, Faculty of Veterinary Medicine, Selcuk University, Konya, Türkiye

## Abstract

Prostaglandins may be one of the important factors affecting the development and quality of the embryo in in vitro production (IVP) and may have an inhibitory effect on the development of the embryo. The supplementation of non-steroidal anti-inflammatory drugs (NSAIDs) in the in vitro culture medium may facilitate embryo development. The objective of this study was to assess the effect of non-steroidal anti-inflammatory drugs on embryo development and quality during bovine in vitro embryo culture. The study involved the performance of a series of procedures, including in vitro embryo production processes, the supplementation of different NSAIDs in the in vitro culture, and the evaluation and staining of embryos. In the initial 3 d of the in vitro culture stage, three different NSAIDs were administered to the groups: flunixin meglumine (FM), meloxicam (Mel), and carprofen (Car). No treatment was applied to the control group at this stage. All groups were incubated in the same culture medium, with the solutions being changed starting from the fourth day of the in vitro culture. On the seventh day of culture, the embryos were evaluated according to their developmental stages and quality. Subsequently, the developing embryos were subjected to differential staining and the TUNEL (terminal deoxynucleotidyl transferase dUTP nick end labeling) method for the assessment of their developmental competence, quality, and apoptotic index. Following the supplementation of NSAIDs in the culture medium, the blastocyst formation rates were found to be higher in the FM, Car, and Mel groups compared to in the control group ( P<0.05 ). Furthermore, the inner cell mass, trophectoderm, and total cell counts were observed to be higher in the FM, Car, and Mel groups in comparison to the control group. In addition, the number of apoptotic cells and the apoptotic index were found to be lower in the FM, Car, and Mel groups compared to the control group. Consequently, it was established that the supplementation of non-steroidal anti-inflammatory drugs in the culture medium during in vitro embryo production resulted in an enhanced rate of blastocyst development and an improved quality of blastocysts.

## 1 Introduction

In vitro embryo production (IVP) is the process of imitating the natural embryo production stages under laboratory conditions. Since many steps are involved during in vitro embryo production, it is reported that there are still unidentified events in this process and that there are many morphological and molecular factors affecting success (Camargo et al., 2006; Hansen, 2020; Hasler, 2000).

It is also stated that prostaglandins may be one of the important factors affecting the development and quality of the embryo in IVP. Several studies have indicated that prostaglandins may have an inhibitory effect on the development of the embryo (Scenna et al., 2004; Scenna et al., 2005; Schrick et al., 1993). Furthermore, Schrick et al. (1993) demonstrated that prostaglandins also negatively impact embryo quality in cattle. Scenna et al. (2004) observed that the presence of prostaglandin in the in vitro culture medium before and during the compact morula had a direct negative impact on embryo development.

It has been observed that embryos that undergo division earlier after fertilization are more likely to reach the blastocyst stage. Embryos of superior quality typically divide for the first time approximately 26 h after fertilization, whereas embryos of inferior developmental competence divide after 36 h. This phenomenon is believed to be attributable to a disparity in the transcriptome profile between early- and late-dividing embryos at the two-cell stage (Lonergan et al., 1999; Patel et al., 2007). Nevertheless, Grycmacher et al. (2019) proposed that the disparity between early- and late-cleavage embryos may be attributed to prostaglandin metabolism. This is because prostaglandin synthetase and prostaglandinF_2*α*_ (PGF_2*α*_) receptor mRNA expression was found to be higher in late-cleavage embryos than in early-cleavage embryos. Nevertheless, it has been demonstrated that the presence of elevated concentrations of prostaglandin during the early stage of the in vitro culture step exerts a detrimental impact on embryo development and quality and, consequently, reduces the blastocyst formation rate (Grycmacher et al., 2019).

Following the identification of the adverse effects of prostaglandins on embryo development, it has been proposed that the supplementation of non-steroidal anti-inflammatory drugs (NSAIDs) in the in vitro culture medium may facilitate embryo development. In studies conducted for this purpose, it has been reported that the supplementation of the non-selective cyclooxygenase (COX) inhibitor flunixin meglumine in the culture medium increases the blastocyst formation rate (Goda et al., 2005; Kim et al., 2014). Although the supplementation of the non-selective COX inhibitor flunixin meglumine in an in vitro culture medium yielded successful results, it was hypothesized that the inhibition of the COX-2 enzyme related to PGF_2*α*_, which has a detrimental impact on early embryo development, using different NSAIDs would be a more effective way of reaching the oocyte to blastocyst stages. Given that flunixin meglumine is not specific to the COX enzyme, it has the potential to impede the synthesis of other prostaglandins that play a role in embryo development. However, the use of selective COX-2 drugs merely inhibits the synthesis of specific prostaglandins. Therefore, the aim of this study was to assess the impact of selective (carprofen and meloxicam) and non-selective (flunixin meglumine) COX-2 cyclooxygenase inhibitor NSAIDs on embryo development and quality during in vitro embryo culture.

## 2 Material and methods

Oocytes were collected from Holstein cattle ( n=155 ) after slaughter in an abattoir. The oocytes were then subjected to maturation, fertilization, and embryo culture under in vitro conditions. Undefined media (BO-Wash, BO-IVM, BO-SemenPrep, BO-IVF, BO-IVC, and BO-Oil) prepared by a commercial company (IVF Bioscience, UK) were used in embryo production (Alkan et al., 2023; Bicici et al., 2023; Najafzadeh et al., 2021).

### 2.1 Collection of oocytes

The study was conducted in six replicates, with a total of 310 ovaries collected from the slaughterhouse. The ovaries were transported to the laboratory within a period of 2–4 h in lactated ringer solution containing 50 µ g mL^−1^ gentamicin. The ovaries were subjected to a cleansing procedure in the laboratory, whereby they were washed on three to four occasions with 0.9 % sodium chloride solution, to remove any blood, tissue residues, and transport medium that may have been present. Subsequently, follicle aspiration was conducted on the ovaries with the assistance of a syringe fitted with an 18 G needle, resulting in the collection of cumulus oocyte complexes (COCs). The collected COCs were scanned under a stereomicroscope, and their morphological characteristics were evaluated (Gordon, 2003). Only category-I (A-quality) COCs were used for maturation in the study.

### 2.2 In vitro maturation and fertilization

Following the identification of COCs, the oocytes were subjected to a series of washes in an oocyte-washing medium (BO-Wash), with each cycle consisting of two to three washes. The oocytes were then transferred into an in vitro maturation medium in four wells that had been previously prepared for this purpose. In the in vitro maturation process, a maximum of 45–50 COCs were placed in each well, and four-well dishes were incubated at 38.5 °C and 5.5 % CO_2_ for 20–22 h following the completion of all oocyte transfers.

Following the maturation process, the degree of cumulus expansion was identified prior to the transfer of the oocytes into an in vitro fertilization medium. Accordingly, cumulus expansion was evaluated in three stages. Grade 1 was defined as exhibiting minimal expansion and morphological change in comparison to the pre-maturation period, grade 2 was characterized by partial cumulus expansion and unexpanded areas, and grade 3 was defined as exhibiting complete or nearly complete cumulus expansion and a homogeneous distribution of cumulus cells (Bicici et al., 2023; Machado et al., 2015). Following this evaluation, only those oocytes exhibiting grade-2 and grade-3 cumulus expansion were transferred into an in vitro fertilization medium (BO-IVF). Subsequently, the semen were prepared for in vitro fertilization. In each application, two to three frozen semen straws were thawed in a 37 °C water bath. The thawed semen were transferred to 15 mL centrifuge tubes containing a semen-washing medium (BO-SemenPrep) and centrifuged at 328×g for 5 min. The supernatant, which remained at the top following centrifugation, was discarded. Subsequently, 2–4 mL of semen-washing medium was added to the tube once more and centrifuged at 328×g for 5 min. The supernatant was then discarded again. The count of spermatozoa (final concentration of $1\times 10^{6}$ mL^−1^) was determined for the remaining portion, and the requisite calculations were made and added to the in vitro fertilization medium containing oocytes. Following this procedure, the oocytes and spermatozoa were incubated overnight at 38.5 °C and 5.5 % CO_2_.

### 2.3 In vitro culture

Prior to the in vitro culture process, a vortex was applied for the denudation of cumulus cells. Thereafter, the presumptive zygotes were transferred into four-well dishes containing an in vitro culture medium (BO-IVC and coated with BO-Oil). The four-well plates were then incubated at 38.8 °C, 6 % CO_2_, 6 % O_2_, and 88 % N_2_. The presumptive zygotes were randomly divided into four groups prior to being placed in a culture medium.

The flunixin meglumine group (FM, n=265 ) received a dose of 5 ng mL^−1^ flunixin meglumine for the first 3 d of the culture period. The FM was dissolved in ultrapure water.

The carprofen group (Car, n=264 ) was supplemented with 3 µ g mL^−1^ carprofen for the first 3 d of the culture period. Carprofen was dissolved in 1:1 DMSO–PBS solution.

The meloxicam group (Mel, n=280 ) was supplemented with 2 µ g mL^−1^ meloxicam for the first 3 d of the culture period. Meloxicam was dissolved in 0.02 M NaOH solution.

For the control group (Con, n=279 ), no supplementation was made in the culture medium.

Embryos were maintained in a culture medium containing NSAIDs for the initial 3 d (FM, Car, and Mel groups). On the fourth day, the culture medium was replaced with a medium devoid of NSAIDs. In the control group, the medium was replaced on the fourth day.

Embryos obtained following culture for a period of 7 to 8 d were evaluated in accordance with the criteria set forth by the International Embryo Technology Society (IETS) (Bó and Mapletoft, 2013).

### 2.4 Determination of cell number of embryos by differential staining

Following the determination of the developmental stages of embryos after in vitro culture, embryos at the blastocyst stage were subjected to the differential staining method (Machado et al., 2015; Maylem et al., 2017). The embryos were initially washed twice in a phosphate-buffered saline (PBS) solution containing bovine serum albumin (BSA) and then were incubated in a solution containing Triton X (100 µ g mL^−1^) for 15 s. Subsequently, the embryos were washed twice with PBS–BSA and kept in a solution containing 0.1 mg propidium iodide (PI) for 15 s. Following the procedure, the embryos were washed and kept in a solution containing Hoechst 33 342 (25 µ g mL^−1^) for 30 min. Finally, the embryos were positioned within a glycerol-based solution on the slide, with a coverslip placed over the top. The slides were visualized under a laser scanning confocal microscope (Nikon A1R1, Japan), and the inner cell mass (ICM), trophectoderm cells (TE), and total cell count (TCC) of the embryos were determined (Alkan et al., 2023).

### 2.5 Determination of cell death and apoptotic index in embryos

The TUNEL (terminal deoxynucleotidyl transferase dUTP nick end labeling) method was used to stain embryos in order to determine the cell death and the apoptotic index in the embryos that had been cultured in vitro (Fouladi-Nashta et al., 2005; Hwang et al., 2013; Paula-Lopes and Hansen, 2002). The embryos, which were removed from the in vitro culture medium and whose developmental stage was determined, were first washed three times in a PBS solution containing polyvinylpyrrolidone (PVP) and then were kept in a 4 % paraformaldehyde solution for 20 min at room temperature. Following the procedure, the embryos were washed three times in PBS–PVP solution. The embryos were then kept in permeabilization solution at room temperature for 10 min and washed three times in PBS–PVP solution. Following the washing procedure, embryos were kept in a terminal deoxynucleotidyl transferase (TdT) equilibration buffer and incubated at 37 °C for 20 min. Then, the embryos were directly transferred to a TUNEL reaction mix and incubated in the dark at 37 °C for 1 h. After this procedure, the embryos were washed three times in a PBS–PVP solution, transferred into DAPI (4^′^,6-diamidino-2-phenylindole), and kept in this solution for 5 min. The embryos were then washed on seven to eight occasions with PBS–PVP solution. Finally, the embryos were transferred to a glycerol solution on a poly-L-lysine-coated glass slide and covered with a coverslip. Following staining, the embryos were examined under a laser scanning confocal microscope (Nikon A1R1, Japan). The apoptotic index was calculated according to Karakas Alkan et al. (2025). The formula is arranged as

$$\begin{array}{rl} & \text{apoptotic index}=\text{count of cells with apoptosis}/\\ & \text{total count of cells}\times 100. \end{array}$$

### 2.6 Statistical analyses

The statistical software SPSS 25.0 (IBM Corp, released 2017, IBM SPSS Statistics for Windows, version 25.0, Armonk, NY) was used for data evaluation. The Kolmogorov–Smirnov test was used to ascertain the normality and homogeneity of the variables' variances. The relationships between categorical variables were analyzed using Fisher's exact test and the chi-square test. In cases where the expected frequencies were below 20 %, the Monte Carlo simulation method was used to incorporate these frequencies into the analysis. The results of normally distributed variables were presented as mean ± standard deviation (SD) and one-way ANOVA post-hoc Tukey honestly significant difference (HSD) test was used to evaluate the variables. The tests were deemed to be statistically significant at the P<0.05 level.

## 3 Results

A total of 2362 COCs of different quality were collected from these ovaries. Throughout the study, only category-I (A-quality) COCs were included in the groups. The mean number of oocytes per ovary (mean ± SD) was 7.61 ± 1.27, and the number of A-quality oocytes was 3.81 ± 0.9. However, the mean numbers of B-, C-, and D-quality oocytes per ovary were 1.63 ± 0.62, 1.42 ± 0.71, and 0.74 ± 0.14, respectively.

**Figure 1:**
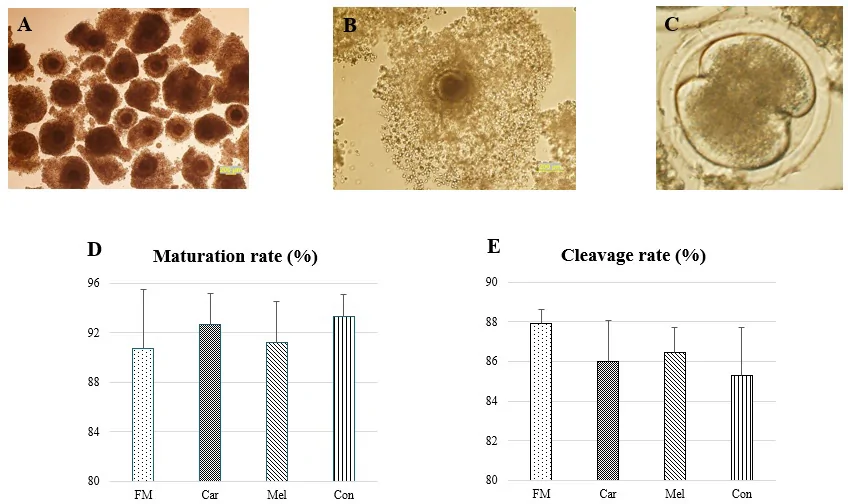
Maturation and cleavage rates of the groups prior to in vitro culture. **(a)** Representative images of selected cumulus oocyte complexes prior to in vitro maturation. **(b)** The degree of cumulus expansion was classified as grade 3 (complete expansion). **(c)** The cleavage-stage embryos and **(d)** the rates of the maturation and **(e)** the cleavage. Abbreviations: FM – flunixin meglumine, Car – carprofen, Mel – meloxicam, Con – control.

Following the assessment of the quality of the COCs, a total of 1183 A-quality oocytes (FM, n=292 ; Car, n=285 ; Mel, n=307 ; Con, n=299 ) were utilized in the study. No statistical difference was detected between the maturation rates of these oocytes according to the groups ( P>0.05 , Fig. 1). The maturation of the oocytes was evaluated by the expansion of the cumulus, and oocytes exhibiting low cumulus expansion (grade I) were not transferred to a fertilization medium. The number of oocytes exhibiting grade-I cumulus expansion was 27, 21, 27, and 20 in the FM, Car, Mel, and Con groups, respectively, and these oocytes were excluded from the study. Furthermore, no statistically significant differences were identified in the cleavage rates of the groups (Fig. 1).

**Figure 2:**
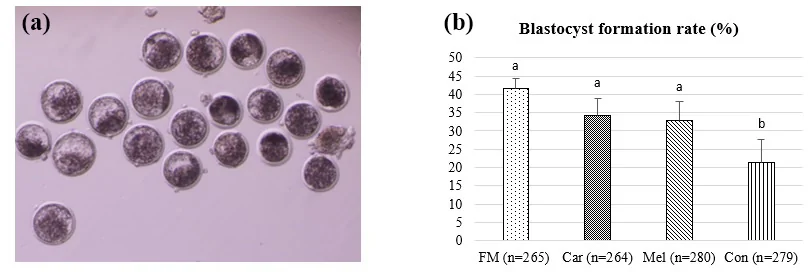
The effect of NSAID supplementation during in vitro culture on the development of bovine embryos. **(a)** Representative images of day-7 IVP embryos and **(b)** blastocyst formation in embryos supplemented with flunixin meglumine (5 ng mL^−1^), carprofen (3 µ g mL^−1^), meloxicam (2 µ g mL^−1^), and 0 (control). ^a,b^ Superscripts represent the statistical difference at P<0.05 . Abbreviations: FM – flunixin meglumine, Car – carprofen, Mel – meloxicam, Con – control.

The blastocyst formation rates are presented in Fig. 2. The blastocyst formation rates were observed to be 41.51 % (110/265) in the FM group, 34.09 % (90/264) in the Car group, 32.86 % (92/280) in the Mel group, and 21.50 % (60/279) in the Con group. Moreover, the blastocyst formation rates were found to be statistically higher in the FM, Car, and Mel groups compared to in the control group. However, no statistically significant difference was observed between the FM, Car, and Mel groups.

**Figure 3:**
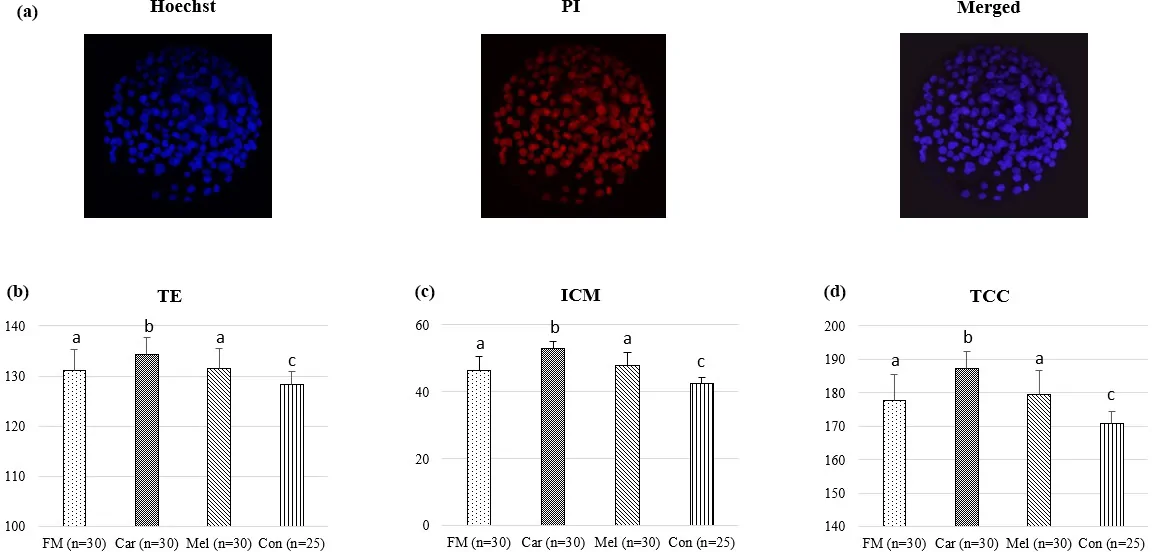
Effect of NSAID supplementation during in vitro culture on ICM, TE, and TCC of bovine embryos. **(a)** Representative images of differential staining of day-7 IVP blastocysts supplemented with flunixin meglumine (5 ng mL^−1^), carprofen (3 µ g mL^−1^), meloxicam (2 µ g mL^−1^), and 0 (control). TE **(b)**, ICM **(c)** numbers, and TCC **(d)** of blastocysts in the indicated groups. Data are presented as the mean ± SD. ^a,b,c^ Superscripts represent the statistical difference at P<0.05 . Abbreviations: ICM – inner cell mass, TE – trophectoderm, TCC – total cell count, IVP – in vitro embryo production, PI – propidium iodide, FM – flunixin meglumine, Car – carprofen, Mel – meloxicam, Con – control.

The cell counts of embryos developing in the groups identified by differential staining are presented in Table 1. The ICM, TE, and TCC were found to be higher in the FM, Car, and Mel groups than in the control group. However, the highest counts of ICM, TE, and TCC were identified in the Car group (Fig. 3). No statistically significant difference was observed between the ICM, TE, and TCC in the FM and Mel groups. Furthermore, the highest ICM / TCC ratio was identified in the Car group. The ICM : TE ratio was the highest in the control group.

**Table 1:** The cell counts of blastocysts obtained from the groups.

Group	ICM	TE	TCC	ICM : TCC (%)	ICM : TE
FM ( n=30 )	46.46 ± 4.17^a^	131.23 ± 4.07^a^	177.70 ± 7.9^a^	26.09	1:2.84
Car ( n=30 )	52.93 ± 2.08^b^	134.23 ± 3.57^b^	187.16 ± 5.09^b^	28.27	1:2.53
Mel ( n=30 )	47.93 ± 3.78^a^	131.56 ± 3.86^a^	179.5 ± 7.11^a^	26.66	1:2.75
Con ( n=25 )	42.56 ± 1.82^c^	128.36 ± 2.64^c^	170.92 ± 3.39^c^	24.89	1:3.02

^a,b,c^ Superscripts represent the statistical difference at P<0.05 . Abbreviations: ICM – inner cell mass, TE – trophectoderm cell, TCC – total cell count, FM – flunixin meglumine, Car – carprofen, Mel – meloxicam, Con – control.

**Figure 4:**
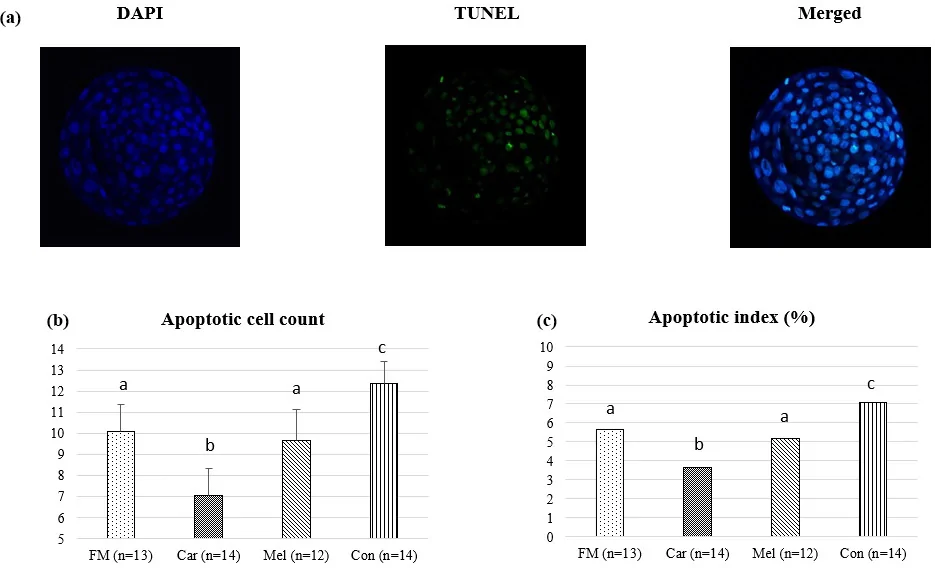
Effect of NSAID supplementation during in vitro culture on apoptotic cell number and apoptotic index of bovine embryos. **(a)** Representative images of TUNEL staining of day-7 IVEP blastocysts supplemented with flunixin meglumine (5 ng mL^−1^), carprofen (3 µ g mL^−1^), meloxicam (2 µ g mL^−1^), and 0 (control). Apoptotic cell numbers **(b)** and apoptotic index ratio **(c)** of blastocysts in the indicated groups. Data are presented as the mean ± SD. ^a,b,c^ Superscripts represent the statistical difference P<0.05 . Abbreviations: DAPI – 4^′^,6-diamidino-2-phenylindole, TUNEL – terminal deoxynucleotidyl transferase dUTP nick end labeling, FM – flunixin meglumine, Car – carprofen, Mel – meloxicam, Con – control.

Apoptotic cell counts and the apoptotic index of embryos are presented in Table 2. The count of apoptotic cells was lower in the groups in which NSAIDs were supplemented in the culture medium compared to in the control group. Furthermore, the apoptotic index was found to be the highest in the control group. However, the count of apoptotic cells was found to be the lowest in the group in which carprofen was supplemented (Fig. 4).

**Table 2:** Apoptotic cell counts and apoptotic index of blastocysts obtained in the groups.

Group	Apoptotic cell count	Total cell count	Apoptotic index (%)
FM ( n=13 )	10.07 ± 1.32^a^	182.23 ± 5.21^a^	5.65 ± 0.55^a^
Car ( n=14 )	7.07 ± 1.26^b^	194.35 ± 3.10^b^	3.63 ± 0.63^b^
Mel ( n=12 )	9.66 ± 1.49^a^	187.58 ± 4.48^a^	5.16 ± 0.85^a^
Con ( n=14 )	12.35 ± 1.08^c^	174.57 ± 6.84^c^	7.08 ± 0.64^c^

^a,b,c^ Superscripts represent the statistical difference at P<0.05 . Abbreviations: FM – flunixin meglumine, Car – carprofen, Mel – meloxicam, Con – control.

## 4 Discussion

A significant number of studies have indicated that prostaglandins may have a detrimental influence on the embryo development and quality (Hockett et al., 2004; Karasahin et al., 2021; Kim et al., 2014; Scenna et al., 2004; Scenna et al., 2005; Schrick et al., 1993). Furthermore, elevated concentrations of PGF_2*α*_ during embryo transfer have been associated with an increased risk of early embryonic loss and implantation failure. Elevated concentrations of PGF_2*α*_ within the uterine lumen have been demonstrated to negatively influence embryo viability (Hockett et al., 2004; Scenna et al., 2004; Scenna et al., 2005; Schrick et al., 1993). Furthermore, it has been reported that the supplementation of PGF_2*α*_ in in vitro culture medium has been found to suppress embryo development in cattle (Scenna et al., 2004). In a study conducted by Kim et al. (2014), the supplementation of PGF_2*α*_ in the culture medium was found to result in a reduction in the rate of hatched blastocyst development and an increase in the count of apoptotic cells. Therefore, in the present study, the effect of the use of different NSAIDs on embryo development and quality was evaluated to eliminate the harmful effects of prostaglandins during in vitro culture of embryos.

There is a dearth of studies utilizing NSAIDs to eliminate the deleterious effects of prostaglandins in bovine IVP. These studies (Goda et al., 2005; Kim et al., 2014) evaluated the effect of only flunixin meglumine supplementation in the in vitro culture medium. In this study, NSAIDs such as carprofen and meloxicam (selective COX-2 inhibitors), which act to inhibit the COX-2 enzyme associated with the inflammatory process, were employed alongside flunixin meglumine. Kim et al. (2014) reported that the supplementation of flunixin meglumine in in vitro culture medium significantly increased the embryo development rate and that the hatched blastocyst development was higher than in the control group. Similarly, Goda et al. (2005) reported that the supplementation of flunixin meglumine (0.005 %) in the co-culture medium increased the rate of blastocyst development and the quality of the blastocysts. Moreover, Razza et al. (2012) demonstrated that the administration of high concentrations of flunixin meglumine at the initial stages of embryo development resulted in a reduction in the rate of blastocyst development. The present study demonstrated that the supplementation of flunixin meglumine, carprofen, and meloxicam during the initial stages of the in vitro culture (the first 3 d) resulted in a higher rate of blastocyst formation compared to in the control group. The obtained results indicate that the suppression of prostaglandin synthesis by inhibiting the COX enzyme in the early period (the first 3 d of culture) may enhance blastocyst development. Nevertheless, the blastocyst formation rate was found to be relatively low in the control group. This is because, in the initial 6 d period following fertilization, a multitude of significant developmental processes take place, beginning with the zygote and culminating in the blastocyst stage. It is therefore evident that the in vitro culture process plays a pivotal role in the development of high-quality blastocysts. At this stage, three key events occur: the first cleavage, which is decisive and highly critical for the subsequent development of the embryo; embryonic genome activation at the 8–16-cell stage; and compacting of the morula on day 5, which marks the initiation of the first cell–cell interaction in the embryo (Hansen, 2020; Lonergan et al., 1999).

The count of cells (ICM and TE) in developing bovine embryos is a crucial parameter in terms of embryo quality and subsequent developmental stages (Carrocera et al., 2016; Maylem et al., 2017; Thouas et al., 2001; Trigal et al., 2011). The ICM is formed by totipotent embryonic stem cells, which are subsequently differentiated into pluripotent epiblast and primitive endoderm cells. The ICM plays a pivotal role in the future development of the embryo. Trophectoderm cells are a precursor to the placenta and the initial component of extra-embryonic structures (Aguila et al., 2022; Brinkhof et al., 2017; Rizos et al., 2008; Watson, 1992). The current study found higher levels of ICM, TE, and TCC in blastocysts developing within the FM, Car, and Mel groups compared to in the control group. Furthermore, the highest counts of ICM, TE, and TCC were observed in the carprofen group. No statistically significant difference was observed between the ICM, TE, and TCC in the FM and Mel groups. In accordance with the findings, it is postulated that the number and quality of cells in the blastocysts developing in the NSAID supplemented groups were increased. Similarly, Goda et al. (2005) and Kim et al. (2014) reported that the supplementation of flunixin meglumine at the in vitro culture stage also improved embryo quality. Besides this, Schrick et al. (1993) and Hockett et al. (2004) have indicated that PGF_2*α*_ has a detrimental effect on embryo quality. Based on this point, it is thought that NSAID supplementation in the culture medium (early period) may eliminate the negative effect of prostaglandins, thereby positively influencing embryo development and quality.

The process of cell death plays a pivotal role in the early embryonic development of mammals. Therefore, a balance between cell proliferation and death is essential for successful embryo development (Ramos-Ibeas et al., 2020). Apoptosis is a type of programmed cell death that is a common feature of mammalian development (Jacobson et al., 1997). The extent of programmed cell death is one of the criteria used to assess embryo development and quality (Kim et al., 2014). It has been demonstrated that various forms of cell death, including apoptosis, may affect the post-implantation development potential of embryos (Gjørret et al., 2003; Gómez et al., 2009; Trigal et al., 2011). Furthermore, the process of embryos reaching the blastocyst stage following fertilization is inherently challenging. The underlying causes of the high prevalence of problems during early embryonal development remain poorly understood (Antunes et al., 2010). Nevertheless, the condensation of chromatin and the presence of fragmented nuclei in these embryos indicate a high rate of apoptosis (Jurisicova et al., 1998; Levy et al., 1998). Apoptosis is an active physiological process that involves chromatin condensation; cell volume reduction; and the formation of apoptotic bodies, which are membrane vesicles (Gjørret et al., 2003; Matwee et al., 2000; Ramos-Ibeas et al., 2020). Consequently, the evaluation of embryonal fragmentation and apoptosis can be employed to ascertain the developmental and qualitative attributes of the embryo (Antunes et al., 2010; Matwee et al., 2000; Paula-Lopes and Hansen, 2002). Kim et al. (2014) reported that blastocysts in the PGF_2*α*_ treatment group exhibited an increased number of apoptotic cells and a significantly elevated level of CASP3 gene expression in comparison to the other groups. However, it was observed that the number of apoptotic cells was reduced in the group to which flunixin had been supplemented (Kim et al., 2014). Indeed, the present study demonstrated a reduction in the count of apoptotic cells in the FM, Car, and Mel groups in comparison to in the control group. This indicates that NSAIDs may be capable of eliminating the apoptotic-stimulating effects of prostaglandins.

In accordance with all of the aforementioned information, the formation rates of blastocyst were observed to be greater in all three groups following the supplementation of non-steroidal anti-inflammatory drugs in the culture medium when compared to the control group. Moreover, when blastocyst formation rates were compared between groups supplemented with flunixin meglumine, carprofen, and meloxicam, no statistically significant differences were observed. However, since the ICM, TE, and TCC of developing blastocysts in the carprofen group were higher than in the other groups and because the apoptotic index rate was lower, it is suggested that supplementing carprofen in the in vitro culture medium may be more beneficial to embryo development.

## 5 Conclusions

It was thus concluded that the supplementation of flunixin meglumine, carprofen, and meloxicam in the culture medium during bovine IVP resulted in a higher rate of blastocyst formation and an increase in the count of cells in the developing embryos. Along with this, NSAIDs have been demonstrated to reduce the count of apoptotic cells. Moreover, it was considered to be the case that the developmental potential of blastocysts (due to the higher cell number and lower apoptotic index ratio) was greater following the supplementation of carprofen in the in vitro culture medium.

## Data Availability

The data set and analyses are available from the corresponding author upon reasonable request.
